# Evaluation of a low-resource screening strategy for ophthalmic pathologies and associated neurological morbidity in an older Tanzanian HIV-positive population

**DOI:** 10.1038/s41598-022-04989-3

**Published:** 2022-01-26

**Authors:** Grace George, Declan C. Murphy, H. D. Jeffry Hogg, Japhet Bright Boniface, Sarah Urasa, Justus Rwiza, Livin Uwemeye, Clare Bristow, Grace Hillsmith, Emma Rainey, Richard Walker, William K. Gray, Stella Maria-Paddick

**Affiliations:** 1 Newcastle University, Newcastle upon Tyne, Tyne and Wear UK; 2 Newcastle upon Tyne Hospitals NHS Foundation Trust, Newcastle upon Tyne, Tyne and Wear UK; 3 Mawenzi Regional Referral Hospital, Moshi, Tanzania; 4 Kilimanjaro Christian Medical University College, Moshi, Tanzania; 5 Northumbria Healthcare NHS Foundation Trust, Newcastle upon Tyne, Tyne and Wear UK; 6 Department of Old Age Psychiatry, Gateshead Health NHS Foundation Trust, Bensham Hospital, Fontwell Drive, Gateshead, Tyne and Wear UK

**Keywords:** Epidemiology, Diagnostic markers, HIV infections, Eye manifestations, Neurological manifestations

## Abstract

Globally, 43 million people are living with HIV, 90% in developing countries. Increasing life expectancy with combination antiretroviral therapy (cART) results in chronic complications, including HIV-associated neurocognitive disorders (HAND) and eye diseases. HAND screening is currently challenging. Our aim was to evaluate clinical utility of retinopathy as a screening measure of HAND in older cART-treated individuals in Tanzania and feasibility of smartphone-based retinal screening in this low-resource setting. A cross-sectional systematic sample aged ≥ 50-years attending routine HIV follow-up in Tanzania were comprehensively assessed for HAND by American Academy of Neurology criteria and received ophthalmic assessment including smartphone-based retinal imaging. HAND and ophthalmic assessments were independent and blinded. Diagnostic accuracy was evaluated by AUROC curves. Of 129 individuals assessed, 69.8% were visually impaired. Thirteen had retinopathy. HAND prevalence was 66.7%. Retinopathy was significantly associated with HAND but HIV-disease factors (CD4, viral load) were not. Diagnostic accuracy of retinopathy for HAND was poor (AUROC 0.545-0.617) but specificity and positive predictive value were high. We conclude that ocular pathology and HAND appear highly prevalent in this low-resource setting. Although retinal screening cannot be used alone identify HAND, prioritization of individuals with abnormal retinal screening is a potential strategy in low-resource settings.

## Introduction

Almost 43 million people are infected by the human immunodeficiency virus (HIV) globally, with 90% of cases in developing countries^1^. In sub-Saharan Africa (SSA), almost 25 million individuals currently live with HIV^2^. Increasing provision of combination antiretroviral therapy (cART) is resulting in both improving life expectancy and emergence of chronic HIV-related complications and chronic diseases as previously seen in high-income countries. HIV-associated neurocognitive disorders (HAND) affect up to 50% of people treated for HIV and are associated with increased morbidity and mortality^3,4^. HAND are classified by severity: asymptomatic neurocognitive impairment (ANI); mild neurocognitive disorder (MND) and; HIV-associated dementia (HAD)^5^. Early identification of HAND and subsequent prompt initiation of treatment, prior to progression of HAND stage, may reduce mortality and reverse neurocognitive deficits^3^. Screening for, and identification of HAND are currently challenging, in SSA and elsewhere. Difficulties include shortage of neurology and psychiatry specialist services in SSA, lack of accurate brief screening measures^6^ and current consensus diagnostic criteria requiring detailed neuropsychological assessment, impractical in busy clinical settings. Additionally, the use of mobile technologies to help address health and human resource shortages in low-resource settings is a recent and expanding area of research and has previously been evaluated for use in remote grading of ophthalmic disease in SSA^7^.

HIV-associated eye diseases occur in up to 70% of HIV positive individuals^8^. Early recognition and treatment can prevent or cure 80% of cases^9^. Features observed in the retina may provide insight about the systemic health of individuals^10^, such as in previous studies where ophthalmoscopy identified biomarkers of hypertension and its severity^11^. Vascular diseases are also common amongst older people living with HIV, and are often undertreated in SSA^12^. There appears to be a strong association between HIV and vasculopathies such as stroke^13^, and renal disease^14^ and retinal disease^15^. Retinal vascular changes has been shown to positively correlate with white matter microstructural changes observed using magnetic resonance imaging in non-HIV positive patients^10^. As white matter disease is also seen in both treated and untreated HIV cases and is associated with HAND^16,17^, we hypothesized that retinal imaging could help identify individuals at risk of HAND.

In this study we aimed to determine the clinical utility of retinopathy as a potential predictive biomarker of HIV-associated neurological complications in older cART-treated HIV positive individuals in Tanzania. A second objective was to determine if retinopathy could be a potential alternative to detailed neuropsychological assessment in a resource poor setting. In addition, we aimed to determine if remote retinal screening using a smartphone based retinal camera was a potentially useful strategy in this context.

## Methods

A cross-sectional study at a single government-funded HIV clinic in Northern Tanzania.

### Participant selection

This study was nested within a larger study of HAND prevalence within the clinic. For the larger study, a systematic sampling methodology was employed. Depending on daily predicted clinic attendance numbers, every second or third routine follow-up patient was systematically selected to take part in the study in order of arrival at the clinic. Participants underwent detailed neurocognitive and clinical assessment. Criteria for inclusion were: ≥ 50-years of age, HIV-positive, attending routine follow up and not acutely unwell. For this ophthalmic sub-study, all recruited participants were offered a subsequent ophthalmic assessment. Due to a wish to avoid participant burden and fatigue, ophthalmic imaging took place at a separate appointment with transport costs refunded and refreshments provided. Those who could not attend, or refused this additional appointment were excluded from the ophthalmic sub-study.

### Informed consent

The purpose for the study and their specific involvement was explained to each potential participant and written informed consent was obtained. Where capacity was in doubt, consent was obtained from close relatives. Ethical approval was obtained from the National Institute for Medical Research (NIMR/HQ/R.8a/Vol. IX/21.36) and Kilimanjaro Christian Medical University College Research Ethics Committee (n.896). This study abides by the tenets of the declaration of Helsinki.

### Assessment

Baseline sociodemographic data of participants, plus HIV disease severity data from standard data sheets, were collected (Table 4 in Appendix). A set of functional assessments were used, including Karnofsky Performance Status^18^, Rockwood Clinical Frailty Scale^19^, and Intervention for Dementia in Elderly Africans (IDEA) low-literacy brief cognitive screen, previously validated for dementia in SSA^20^. Hypertensive and diabetic status were recorded. We did not ask about illicit drug use for this study, as our previous study in this same older age group from the same clinic (n = 253) found no participant reported illicit drug use on the MINI screen and local clinicians advised this question to be inappropriate in an older population^21^. Alcohol history was obtained as previous studies did report alcohol use in this population. Visual acuity (Va) was assessed with available correction using the Landolt broken-ring low-literacy “C” chart with notations for testing at 3 m due to unknown rates of illiteracy amongst the study participants. Visual fields were tested using confrontation (quadrant finger counting) and colour vision with Ishihara charts.

All participants underwent direct ophthalmoscopic examination following pupil dilatation (1% tropicamide) and retinal images were obtained using the iNview retinal camera (VOLK, Cleveland, USA). This retinal camera attached to an iPhone 6 (8-megapixel iSight camera with 1.5µ pixels) was used throughout the study. The red-light reflex was observed and maintained throughout approaching the patient until focused on the retina, much like fundoscopy. Images were then taken automatically by the retinal camera. Images were independently reviewed by 3 ophthalmologists, 2 of whom were based in Tanzania (JR, LU) and 1 from the UK (JH).

### HAND assessment and diagnosis

HAND was diagnosed by consensus using AAN 2007 gold standard criteria^5^ based on neuropsychological battery measures validated for cross-cultural use by the WHO, clinical/neurological examination findings, to determine if other psychiatric disorders better explained cognitive performance, and a collateral history from a close relative or friend. All participants underwent additional detailed locally-validated low-literacy neuropsychological tests of cortical function, the details of which have previously been published^6^. Domains assessed included working memory, verbal memory (learning, delayed recall and recognition memory), fine motor control, motor speed, visuoconstruction, executive function and comprehension. Data were collected in a separate room by research assistants and nurses who had been specifically trained, had previous experience and an annual update and harmonization of methods process lasting one week. Research assistants were blinded to whether patients had retinopathy. Practice trials and errors were managed according to a standardized protocol and tests selected were validated locally and/or in low literacy SSA settings. Neuropsychological test results were compared to performance means for those with and without 4 years of formal education generated in 2016 from a comparison group of similar age and education level attending the ophthalmology outpatient department of MRRH^6,21^. The collateral history and all clinical and neuropsychological information was used for a multi-disciplinary discussion of each participant by the research team to confirm diagnoses and record a detailed case summary. Diagnoses were confirmed or refuted by consensus panel of specialists in old age psychiatry with experience of HAND diagnosis.

### Ophthalmologist criteria for defining eye disease

The definitions for visual impairment used followed those given in the National Sensory Impairment Partnership’s Framework^22^. Mild-moderate visual impairment was defined as LogMAR 0.3 < Va ≤ 0.8 and severe visual impairment was defined as LogMAR Va > 0.8. “glaucoma suspect” was termed if cup-to-disc ratio was > 0.6^23^. A Tanzania-based ophthalmology trainee (JR) determined whether glaucoma could be diagnosed based on ICD-10 criteria^23^. Retinopathy was assumed if any of the following features were observed upon ophthalmoscopy: retinal haemorrhages; microaneurysms; areas of capillary non-perfusion; or cotton wool spots.

### Statistical analysis

All statistical analyses were performed using IBM SPSS software V24.0. Normally distributed data were described using mean, standard deviation (SD) and 95% confidence intervals (CI). To assess the independent influence of age, gender, HIV severity, functional status, HAND severity and visual change on retinopathy status, univariate and multivariable logistic regression models were developed with retinopathy as the dependent (outcome) variable. Comparisons were made using independent t-tests. Non-normally distributed data were described using median, interquartile range (IQR) and frequency and compared using Mann–Whitney U test. Categorical variables were described using frequency and compared using chi-squared test and with the Fisher’s exact test correction where appropriate. The statistically significant variables were then considered as independent variables in a multivariable model examining their relationship to the outcome. A stepwise, backwards approach to model building was employed, based on their likelihood ratio. Only significant variables were retained in the final model. The validity and robustness of the model was checked by examining studentized residuals, eigenvalues and the tolerance. Statistical significance was defined as *P* ≤ 0.05.

The performance of the smartphone HIV retinopathy screen was investigated using Area Under the Receiver Operating Characteristic (AUROC) curve analysis. Diagnostic accuracy was analysed for HAND and symptomatic HAND (s-HAND, MND/HAD). To determine diagnostic accuracy for HAND, ANI/MND/HAD were coded 1 and all others coded 0. For S-HAND, MND/HAD were coded 1, and all others 0. Diagnostic accuracy was also analysed for HIV stage 3 or 4 coded as 1 and all others coded 0 as well as analysed for detectable viral load coded as 1 and undetectable as 0. When grouped together, the predictive value retinopathy to determine late HIV stage, detectable viral load as well as HAND and s-HAND was analysed.

### Ethics approval and consent to participate

The purpose for the study and their specific involvement was explained to each potential participant and written informed consent was obtained. Where capacity was in doubt, consent was obtained from close relatives. Ethical approval was obtained from the National Institute for Medical Research (NIMR/HQ/R.8a/Vol. IX/21.36) and Kilimanjaro Christian Medical University College Research Ethics Committee (n.896).

## Results

### Population characteristics

**Figure 1:**
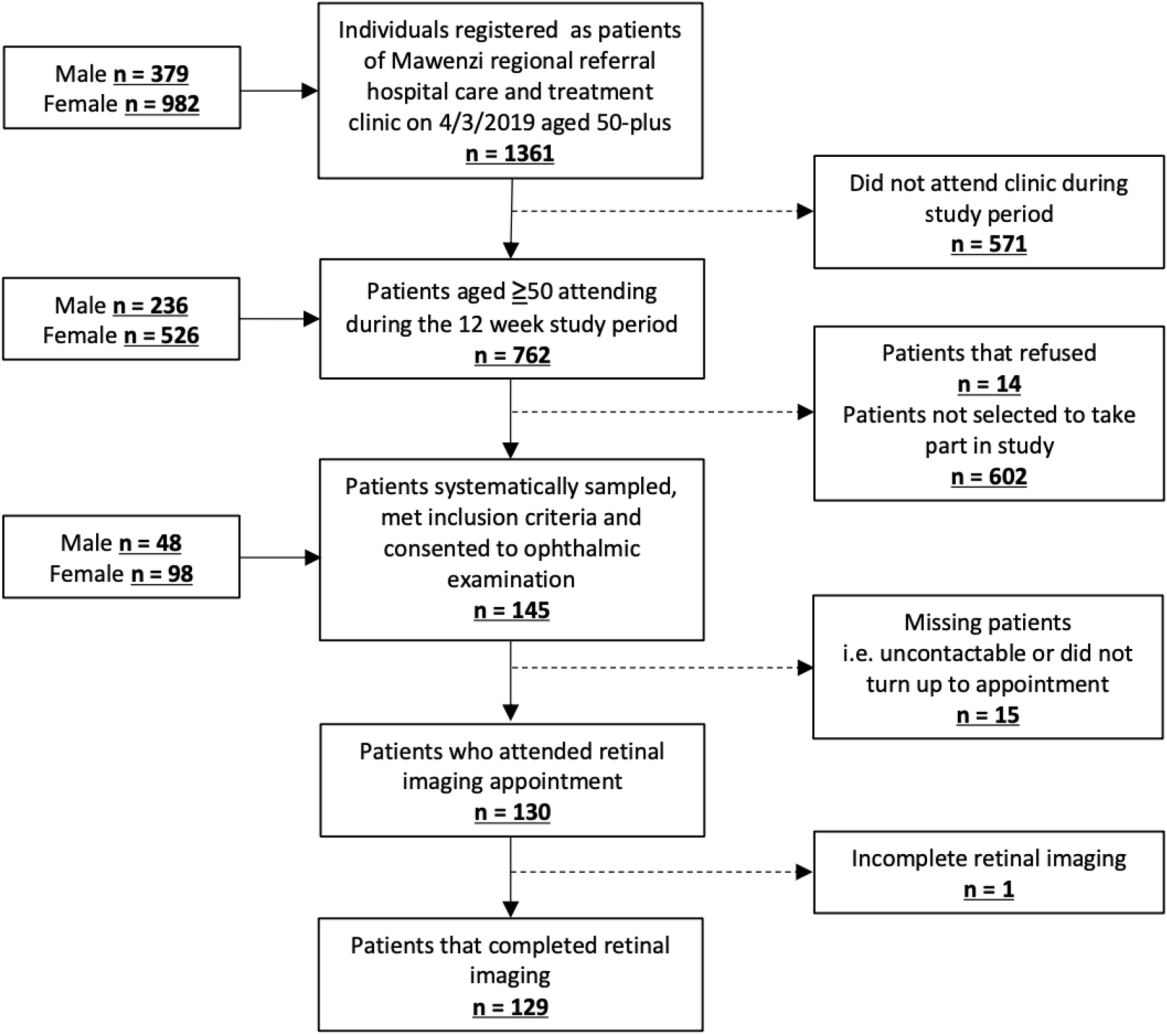
Flowchart showing study participants and those excluded.

Of the 762 individuals aged ≥ 50 years attending follow-up during the study period, 145 were systematically sampled and consented to inclusion. Of these, 129 completed the ophthalmologic assessment and had complete data (Fig. 1). One was unable to complete imaging due to discomfort. There was no significant difference in age, CD4 count or viral load between those who had complete data and those who did not.

Median patient age was 56 years (IQR: 53–61), 83 (64.3%) were female, 88 (68.2%) self-reported at least 5–7 years of primary-school education and 8 (6.2%) were illiterate. Median time since HIV diagnosis was 10.5 years (Range: 3 months to 23 years) and median nadir CD4 count was 168 cells/mm^3^ (IQR: 106–251). All but one participant was receiving antiretroviral treatment when enrolled in the study. HIV-disease appeared well managed with current median CD4 434 cells/mm^3^ and 76% with suppressed HIV viral load (defined as < 20 copies/ml). Twenty-one (16.3%) reported a prior hypertension diagnosis, 11 (52.4%) of whom were on anti-hypertensives. Thirty-eight (29.5%) of patients were hypertensive in clinic (blood pressure (BP) > 140/90 mm of mercury (mmHg). The mean BP in clinic was 134.5/81.2 mmHg with a maximum systolic of 219 mmHg. Seven (5.4%) patients reported being diabetic, 5 (71.4%) of whom were on treatment.

Forty-three patients (33.3%) did not meet HAND criteria, 41 (31.8%) met criteria for ANI, 43 (33.3%) met criteria for MND and 2 patients (1.6%) were diagnosed with HAD.

### Overall prevalence of ophthalmic disease

Recent visual changes were self-reported by 71 patients (55%), but none reported having sought ophthalmological review.

Fifty-three patients (41.1%, 95% CI 32.6–49.6) had mild-moderate visual impairment (0.3 < Va ≤ 0.8) and 37 (28.7%, 95% CI 20.9–36.5) severe visual impairment (Va > 0.8). Six (4.7%) had a visual field defect, 4 (3.1%) cataracts and 5 (3.9%) abnormal colour vision. The cataracts were not considered too dense to prevent visualization of the retina. Fifty-eight patients (45%) had Va > 0.6 and abnormal fundal signs were seen in 53 patients (41.1%), including 16 (12.4%) referred for suspected glaucoma and 6 (4.7%) for suspected HIV retinopathy. One showed signs consistent with cytomegalovirus retinitis. Once reviewed by an ophthalmology specialist (JH), retinopathy was suspected in 13 patients (10.1%) and glaucoma was suspected in 19 patients (14.7%) (CDR > 0.6) but following diagnostic assessments only one patient was diagnosed with bilateral primary angle closure glaucoma. Sixty-seven patients (51.9%) were referred for full ophthalmic assessment.

### HIV retinopathy and its association with HAND

Features of retinopathy were significantly associated with both HAND diagnosis and HAND severity (Table 1). Retinopathy was associated with patient-reported vision loss during the previous 3 months (χ^2^(1, n = 129): 5.111; p = 0.037).

**Table 1:** Comparison of characteristics between patients with suspected human immunodeficiency virus retinopathy and those without: univariate logistic regression model with dichotomised presence of suspected HIV retinopathy as the dependent (outcome) variable.

Variable	Patients with HIV retinopathy (n = 13)	Patients without HIV retinopathy (n = 116)	Significance
Age (median (IQR)), N = 129	60 (54.5–67.5)	56 (53–61)	U = 612.5 Z = − 1.309 p = 0.191
**Gender, N = 129**			
Female	7 (53.8%)	76 (65.5%)	X^2^ = 0.694 p = 0.543
Male	6 (46.2%)	40 (34.5%)	
CD4 count (median (IQR)), N = 114 (15 missing values)	474 (293–551)	434 (258–684)	U = 558.0 Z = − 0.088 p = 0.930
**Viral load (frequency), N = 105 (24 missing values)**			
Detectable	5 (38.5%)	39 (33.6%)	X^2^ = 0.298 p = 0.739
Non-detectable	5 (38.5%)	56 (49.3%)	
Karnofsky Performance Status (median (IQR)), N = 129	90 (80–100)	100 (90–100)	U = 555.0 Z = − 1.787 p = 0.074
Clinical Frailty Scale (median (IQR)), N = 129	2 (2–3)	2 (2–3)	U = 558.5 Z = − 1.727 p = 0.084
**HAND diagnosis (frequency), N = 129**			
No HAND	2 (15.4%)	41 (31.8%)	U = 510.0 Z = − 2.051 p = 0.04
ANI	3 (23.1%)	38 (29.5%)	
MND	7 (53.8%)	36 (27.9%)	
HAD	1 (7.7%)	1 (0.8%)	
**Subjective change in vision (frequency), N = 129**			
Yes	11 (84.6%)	60 (51.7%)	X^2^ = 5.111 p = 0.037
No	2 (15.4%)	56 (48.3%)	

Using logistic regression, HAND severity and self-reported visual change were assessed for significance against retinopathy. The significance intervals, odds ratios and confidence intervals are shown in Table 2. This shows HAND severity is associated with retinopathy, adjusting for co-variates (Nagelkerke R^2^ = 0.197, Hosmer and Lemeshow goodness of fit, X^2^ (4) = 0.647, p = 0.958).

**Table 2:** Multivariable logistic regression model with dichotomised suspected HIV retinopathy as the dependent (outcome) variable.

Variable	Odds ratio (95% CI)	Significance (p)
HAND severity	2.406 (1.117–5.184)	0.025*
Subjective change in vision	0.216 (0.044–1.075)	0.061

### Diagnostic accuracy

The predictive value of retinopathy for HAND, s-HAND, late HIV stage and detectable viral load are presented in Table 3. Overall diagnostic accuracy was poor. AUROC ranged from 0.545–0.617, with 95% CI spanning 0.5 for each variable. Specificity was high throughout whereas sensitivity was low. Positive predictive value (PPV) was high for HAND and HIV stage 3 whereas PPV for s-HAND and detectable VL was low. Of all the screening options evaluated, the highest screening accuracy was achieved for HAND using retinopathy screen (AUROC = 0.617, sensitivity 13.4%, specificity 95.7% and PPV 0.846).

**Table 3:** AUROC for relation of variables to HIV retinopathy.

	AUROC (95% CI)	Sensitivity	Specificity	Positive predictive value	Negative predictive value
HAND (n = 82)	0.617 (0.471–0.763)	0.134	0.957	0.846	0.388
Symptomatic HAND (n = 27) (MND/HAD)	0.597 (0.424–0.771)	0.185	0.922	0.385	0.81
HIV Stage 3/4 (n = 105)	0.557 (0.404–0.711)	0.144	0.957	0.923	0.191
Detectable VL (n = 44)	0.545 (0.355–0.734)	0.144	0.918	0.5	0.589

## Discussion

Our findings suggest that retinopathy may be associated with HAND in older adults receiving long term HIV treatment in this setting. However, identification of retinopathy does not appear to be a useful strategy for identifying those with HAND, or those with frailty. AUROC was not performed on clinical frailty scale as there was no statistically significant association with retinopathy. The high specificity of retinopathy screening may have implications for clinical practice because retinal imaging may show potential to ‘rule in’ which individuals with HIV are likely to be at risk of cognitive impairment. Those negative for retinopathy, have a higher probability of not meeting HAND criteria (normal cognition). In addition, a negative retinopathy assessment does not reliably exclude HAND. This means that retinopathy screening cannot be used alone to determine who might need assessment for HAND. However, retinal screening could allow staff to prioritize individuals with retinal signs, due to high PPV.

Although the relationship between retinopathy and HAND is unclear, there are commonalities in the pathogeneses of both HIV-related complications which may explain their relationship. HIV retinopathy most likely occurs secondary to microvasculopathy from either immune complex deposition, increased plasma viscosity or vascular endothelium invasion by HIV, opportunistic infections or malignancy^24^. HAND occurs through a number of mechanisms including opportunistic central nervous system (CNS) infections, direct neurotoxic effect of the HIV virus and neurotoxic effect of cART^4^. Contributory factors include HIV disease severity, accelerated vascular disease and frequent co-morbidities^4^.

The World Health Organization (WHO) advocates that screening for mental disorders be integrated into chronic disease monitoring to address the shortage of specialist personnel in low-resource settings^25^. Eye disease is currently a major cause of morbidity and disability in both HIV and non-HIV populations and disproportionately affects older people^26^. Cataracts, glaucoma, uncorrected refractive error and trauma are the major reported causes of visual impairment^26^. Identification and screening for ophthalmic disease in low-resource settings is currently challenged by similar issues to those noted for chronic complications of HIV. These include lack of specialist personnel, particularly in rural and remote areas, a focus on acute intervention rather than primary care and prevention, lack of integrated referral systems and access to affordable necessary equipment^27,28^.

Using mobile technologies to help address health and human resource shortages in low-resource settings is a recent and expanding area of research and is increasingly feasible given the rapid increase in smartphone use across SSA^29^. Low-cost smartphone applications have been evaluated for use in remote grading of ophthalmic disease in SSA^7^. Non-clinical imagers were able to capture images at a standard that enabled remote grading at the level of a desktop retinal camera^7^. In some regions of SSA there is only one ophthalmologist per 2.5 million people, so remote retinal imaging could ameliorate the impact of understaffing^30^.

In this study, we demonstrate a high prevalence of visual impairment in our study participants of HIV-positive patients, with 41.1% having mild-moderate visual impairment and 28.7% severe visual impairment. A previously published study reported that 11.2% of HIV-positive individuals aged 18 years or greater had a visual acuity of 0.2 or more in at least one eye^31^. One previous study in HIV-negative individuals aged 50 or greater report visual impairment of 13.6% in SSA^32^. Our prevalence was much higher. This difference may relate to our study participants being 50 years or older whereas other studies investigated younger sample. In addition, other explanations may include a risk of false positives from unidentified refractive causes of acuity loss, a lower threshold for diagnosis of visual impairment or our small sample size^31^. Factors relating to the willingness of patients to present with ophthalmic features may also contribute, perhaps due to lack of symptom knowledge, cost or impaired cognition. Since there is a high prevalence of eye disease in this study population, it is prudent to screen for it.

We suspect the high prevalence of visual impairment may be seen also in the background local population, due to lack of routine eye tests and low patient presentation, even when high quality, affordable services are available^33^. Rates of visual impairment in the remote and rural areas may in fact be worse than those regularly attending hospital due to cost and availability of transport. In addition, low-cost retinal imaging may enable the effective monitoring of patients with HIV-associated ophthalmic diseases and could address challenges in healthcare provision relating to the limited availability of healthcare professionals in these regions^30^.

### Strengths and limitations of the study

To our knowledge, this is the only study on HIV retinopathy in Tanzania, thus providing unique data on visual impairment and ocular disease prevalence. Previous studies looking at disease amongst HIV-infected individuals in SSA, excluded patients with confusion or altered mental state^31^. Since this study was able to assess the link between HIV ophthalmic diseases and HAND, this was an additional strength.

There are several limitations to this study. Images obtained from VOLK iNview retinal camera were of low-to-moderate quality so subtle changes or more complex pathologies may have been missed. Therefore, there is a substantial reliance on external referrals to ensure a correct diagnosis is made.

There are non-HIV causes of the fundal signs used to define retinopathy, such as hypertensive and diabetic retinopathy. A large proportion of our study participants had a blood pressure over 140 mmHg, however ‘white-coat effect’ is relatively common in SSA^34^. Seven of the 13 (53.8%) suspected HIV retinopathy patients had hypertension in clinic; we cannot exclude hypertensive retinopathy in these individuals. Despite individuals not self-reporting as diabetic and being under regular clinic follow-up, we found that 9 patients had glycosuria, a clinical sign of undiagnosed or untreated diabetes. 5 of these 9 had retinal signs thought to be consistent with HIV retinopathy but without measuring HbA1c, we cannot exclude diabetic retinopathy in these individuals.

In addition, dementia and cognitive impairment might have in fact been due to other causes, such as vascular dementia, Parkinson’s disease dementia or alcohol related. To minimize this, however, the consensus diagnosis process considered these alternative differential diagnoses, taking into account the neurological examination, history, risk factors and cognitive profile. Those diagnosed with HAND met HAND criteria, following consensus panel review.

This research only relates to HIV positive adults aged 50-years or older. This may be a strength in that studies of this older cART-treated population are few in SSA, but limits generalizability.

## Conclusions and recommendations for future work

Ocular pathology and HAND were both common in this patient population. We demonstrate that retinopathy is associated with the presence of HAND. This has implications for clinical practice as retinal imaging may have ‘rule in’ value in predicting which individuals with HIV are likely to be at risk of cognitive impairment and so could be used as a potential alternative to detailed neuropsychological assessment. Substantial clinical need for low-resource screening tools for HAND has been identified and although retinal screening cannot be used alone to determine who might need assessment for HAND, it may offer healthcare professionals a tool to enable patient prioritization and so therefore using a smartphone based retinal camera may potentially be a useful strategy in this context.

As early diagnosis of ocular pathology is crucial to allow for timely intervention, portable and affordable tools which image the retina and enable remote diagnosis and monitoring of ophthalmic conditions in HIV-positive individuals. As technology develops, such assessments will become more accurate and useful.

## Data Availability

The datasets used and/or analysed during the current study are available from the corresponding author on reasonable request.

## Appendix

See Table 4.

**Table 4:** Table of baseline participant data collected.

Baseline data	Age, gender, marital status, current weight, current height, hip to waist ratio, smoking status, level of physical activity
Past medical history	Previous stroke, previous diagnosis of hypertension (plus whether on treatment and if adequately controlled), headaches and description of headaches, central nervous system infection in last 12 months and previously, Tuberculosis, weight loss, serious life events in past year, geriatric depression score
Functional assessments	Barthel index, Karnofsky performance status, Rockwood clinical frailty scale, neuropsychological assessment
socioeconomic data	Level of education, occupation, retirement status
Clinical investigations	Blood pressure, electrocardiogram, ankle-Brachial Pressure Index, bioimpedance, urine sample
HIV severity data	Date diagnosed, time since diagnosis, nadir CD4, CD4 count, Viral load (in last 12 months), WHO clinical stage (from notes and reviewed and diagnosed by us)
Blood tests	CD4 count, viral load, full blood count, cholesterol, albumin, creatinine, urea, hepatitis B, hepatitis C, syphilis
Ophthalmic assessments	Experience of change in vision, visual acuity, visual field defect, visual inattention, nystagmus, fundoscopy, cataracts seen, retinal imaging

## Abbreviations

ANI: Asymptomatic neurocognitive impairment
AUROC: Area under receiver operating characteristic
BP: Blood pressure
CI: Confidence intervals
CNS: Central nervous system
HAD: HIV-associated dementia
HAND: HIV-associated neurocognitive disorder
HIV: Human Immunodeficiency Virus
IDEA: Intervention for dementia in elderly Africans
IQR: Interquartile range
mmHg: Millimetres of mercury
MND: Mild neurocognitive disorder
PPV: Positive predictive value
SD: Standard deviations
SSA: Sub-Saharan Africa
s-HAND: Symptomatic HAND
Va: Visual acuity
WHO: World health organisation
